# Improving adeno-associated viral (AAV) vector-mediated transgene expression in retinal ganglion cells: comparison of five promoters

**DOI:** 10.1038/s41434-022-00380-z

**Published:** 2023-01-13

**Authors:** Bart Nieuwenhuis, Elise Laperrousaz, James R. Tribble, Joost Verhaagen, James W. Fawcett, Keith R. Martin, Pete A. Williams, Andrew Osborne

**Affiliations:** 1 John van Geest Centre for Brain Repair, Department of Clinical Neurosciences, University of Cambridge, Cambridge, UK; 2 Cambridge Institute for Medical Research, University of Cambridge, Cambridge, CB2 0XY UK; 3 Department of Clinical Neuroscience, Division of Eye and Vision, St. Erik Eye Hospital, Karolinska Institutet, Stockholm, Sweden; 4 Laboratory for Regeneration of Sensorimotor Systems, Netherlands Institute for Neuroscience, Royal Netherlands Academy of Arts and Sciences (KNAW), Amsterdam, The Netherlands; 5 Centre for Neurogenomics and Cognitive Research, Amsterdam Neuroscience, Vrije Universiteit Amsterdam, Amsterdam, The Netherlands; 6 Centre of Reconstructive Neuroscience, Institute of Experimental Medicine, Prague, Czech Republic; 7 Centre for Eye Research Australia, Royal Victorian Eye and Ear Hospital, Melbourne, VIC Australia; 8 Ophthalmology, Department of Surgery, University of Melbourne, Melbourne, VIC Australia; 9 Ikarovec Ltd, The Norwich Research Park Innovation Centre, Norwich, UK

**Keywords:** Visual system, Genetic transduction

## Abstract

Recombinant adeno-associated viral vectors (AAVs) are an effective system for gene transfer. AAV serotype 2 (AAV2) is commonly used to deliver transgenes to retinal ganglion cells (RGCs) via intravitreal injection. The AAV serotype however is not the only factor contributing to the effectiveness of gene therapies. Promoters influence the strength and cell-selectivity of transgene expression. This study compares five promoters designed to maximise AAV2 cargo space for gene delivery: chicken β-actin (CBA), cytomegalovirus (CMV), short CMV early enhancer/chicken β-actin/short β-globulin intron (sCAG), mouse phosphoglycerate kinase (PGK), and human synapsin (SYN). The promoters driving enhanced green fluorescent protein (eGFP) were examined in adult C57BL/6J mice eyes and tissues of the visual system. eGFP expression was strongest in the retina, optic nerves and brain when driven by the sCAG and SYN promoters. CBA, CMV, and PGK had moderate expression by comparison. The SYN promoter had almost exclusive transgene expression in RGCs. The PGK promoter had predominant expression in both RGCs and AII amacrine cells. The ubiquitous CBA, CMV, and sCAG promoters expressed eGFP in a variety of cell types across multiple retinal layers including Müller glia and astrocytes. We also found that these promoters could transduce human retina ex vivo, although expression was predominantly in glial cells due to low RGC viability. Taken together, this promoter comparison study contributes to optimising AAV-mediated transduction in the retina, and could be valuable for research in ocular disorders, particularly those with large or complex genetic cargos.

## Introduction

Recombinant adeno-associated viral (AAV) vectors can achieve efficient neuronal transduction in the central nervous system (CNS). They exhibit low immunogenicity [1–3] and mainly initiate transgene expression by forming episomal DNA in the nucleus of transduced cells [4]. These factors are key to why AAVs are frequently used in clinical trials in ophthalmology and neurology [5–9]. To date, over 30 AAV clinical trials have commenced for ocular diseases in which most are targeted for monogenic, Mendelian retinal diseases [5–9]. AAV2 is the most widely used serotype due to efficient transduction of retinal neurons [10–12], superior safety over lentiviral vectors [13], effective transduction in injured [14] or diabetic eyes [15], and long-term transgene expression [16–19]. AAV2 is also the serotype in Luxturna, the first ocular AAV gene therapy to obtain regulatory approval in the United States and Europe [20].

The biology of AAV is well understood and it is becoming apparent that several factors are crucial for their transduction efficiency: (1) the AAV capsid; (2) the promoter type; (3) the method of administration; (4) the animal species; and (5) the vector preparation quality [21]. The AAV2 genome has a maximum cargo capacity of ~4.7 kilobase [22, 23] and therefore the size of DNA regulatory elements and the transgene are important considerations for efficient AAV vector design. The promoter regulates the level of transgene expression and determines cell-specificity, as the activation state of the promoter is dependent on the transcription factor machinery present in the transduced cells. There has been great interest in identifying promoters for optimal transduction of different cell types within the retina [14, 24–43] and an ideal promoter would preferably be small, to make the incorporation of large and complex cargos possible.

Retinal ganglion cells (RGC) are the output neurons of the retina with their axons making up the optic nerve. RGCs are primarily affected in ocular neurodegenerative diseases such as glaucoma, diabetic retinopathy and mitochondrial optic neuropathies. The specific targeting of RGCs, with minimum off-target transduction, could be important for the delivery of cell autonomous transgenes, and is particularly interesting for studies aiming to promote axon regeneration after optic nerve injury. In contrast, a broad cellular tropism may be favourable for studies aiming to deliver secreted growth- and survival factors to the retina.

This study compares five promoters in AAV2. The chicken β-actin (CBA), cytomegalovirus (CMV), short CMV early enhancer/chicken β-actin/short β-globin intron (sCAG), mouse phosphoglycerate kinase (PGK) and human synapsin (SYN) promoters were investigated as they are relatively small and commonly used for exogenous expression in the nervous system. Each promoter was linked to the fluorescent reporter enhanced green fluorescent protein (eGFP) so that the strength of transgene expression and cell-specificity could be assessed in the mouse retina, 4 weeks after intravitreal delivery (a typical time point for gene therapy experiments in rodent systems). Furthermore, the viral vectors were preliminarily tested on human post-mortem retinal explants to compare their translational potential. This comparison study helps clarify transduction differences between commonly used promoters and aids the advancement of AAV2-mediated gene transfer in the inner retina.

## Materials and methods

### AAV vector plasmids

**Fig. 1:**
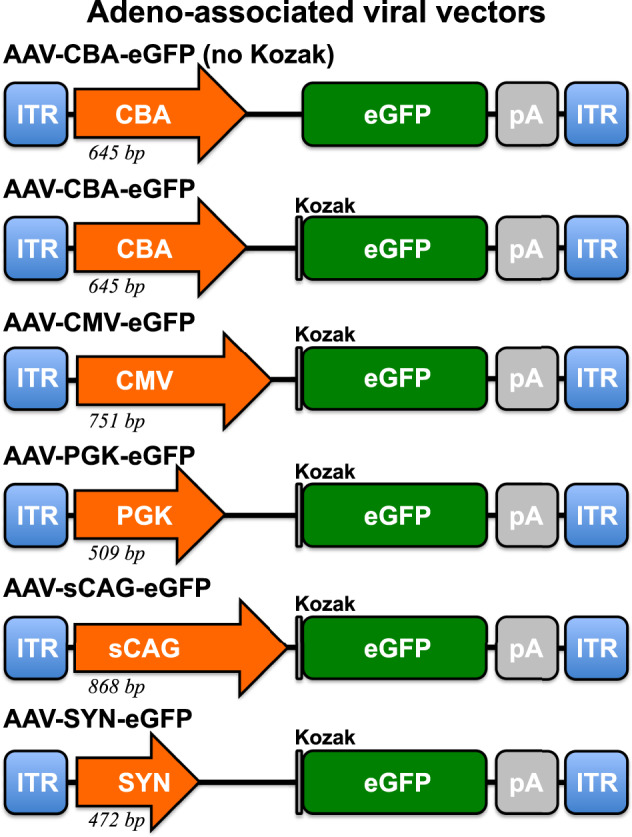
Schematic representation of adeno-associated viral vectors that express enhanced green fluorescent protein (eGFP, 720 bp) under different promoters. Vectors have the Kozak consensus sequence (GCCACCATGG) surrounding the start codon of eGFP, if indicated by the grey vertical line. A polyadenylation signal is located downstream of the transgene in all investigated vectors. The packaging cassettes are flanked by inverted terminal repeats (ITR). The investigated viral vectors did not have chimeric introns or a woodchuck hepatitis virus posttranscriptional regulatory element (WPRE) for promoter comparison purposes and to make incorporation of potential large transgenes possible. bp base pair, pA polyadenylation signal.

AAV-CBA-eGFP (no Kozak) was made by removing the WPRE sequence from a plasmid provided by the Keith Martin laboratory. AAV-CBA-eGFP was made by adding the sequence 5’ GCCACC 3’ directly upstream of the start codon of eGFP in above-mentioned plasmid via site-directed mutagenesis (GenScript Biotech, 860 Centennial Ave, Piscataway, NJ 08854, USA). AAV-sCAG-eGFP was provided by the laboratory of Joost Verhaagen [44, 45]. AAV-CMV-eGFP (Addgene plasmid #193022) was made by removing the β-globin intron upstream of the eGFP sequence from a plasmid that was gifted from Connie Cepko (Addgene plasmid #67634). AAV-PGK-eGFP (Addgene plasmid #193023) was made by removing the β-globin intron preceding eGFP from one of our plasmids (Addgene plasmid #162513) [45]. AAV-SYN-eGFP (Addgene plasmid #162512) is described previously [45]. DNA sequences for the promoters are provided in Supplementary Table 1 and construct schematics in Fig. 1. The AAV vector plasmids were validated for the presence of two inverted terminal repeats (ITRs) (Supplementary Fig. 1) and transgene expression in vitro (Supplementary Fig. 2) prior to AAV preparation.

### Adeno-associated viral vector preparation

The plasmids were packaged into AAV vector serotype 2 by Vigene Biosciences (9430 Key West Avenue, Suite 105, Rockville, MD 20850, USA) utilising standard plasmid transfection protocols and AAVs were extracted from human embryonic kidney 293T (HEK293T) cells via repeated freeze-thawing and iodixanol gradient ultracentrifugation. Vectors were titred by SYBR Green quantitative polymerase chain reaction with primers to the ITRs and suspended in PBS/0.001% pluronic F68 surfactant. An additional titre verification was performed using a QuickTiter^TM^ AAV quantification kit (#VPK-145; Cell Biolabs) after the AAV vectors were diluted and titre matched. The production and validation titre for each AAV2 is shown in Supplementary Table 2 with all vectors used after a single freeze thaw.

### HEK293T plasmid transfection

HEK293T cells were cultured in poly-L-lysine (10 µg/ml, Sigma-Aldrich) coated 6-well plates in Dulbecco’s minimum essential medium (DMEM) containing 10% foetal bovine serum, 1% penicillin/streptomycin until 80% confluent. Medium was then exchanged for DMEM (no additives) and cells transfected with 4 µg plasmid DNA and 4 µl/ml lipofectamine (Thermo Fisher Scientific) for 48 h at 37 °C prior to imaging.

### Mice and intravitreal injection of AAV vectors for transgene delivery to the retina

All animal work was carried out in accordance with the UK Home Office Regulations for the care and use of laboratory animals, the UK Animals (Scientific Procedures) Act 1986 Amendment Regulations 2012 following ethical review by the University of Cambridge Animal Welfare and Ethical Review Body. Individual study protocols were approved by Stockholm’s Committee for Ethical Animal Research (10389-2018). Animal work also met the requirements by the Association for Research in Vision and Ophthalmology’s Statement for the Use of Animals in Ophthalmic and Visual Research.

Thirty 8-week old male C57BL/6J mice (Charles River Laboratories) were used for transduction comparisons. Bilateral, intravitreal injections were performed under general anaesthesia (50 mg/kg ketamine and 10 mg/kg xylazine) with 0.4% oxybuprocaine hydrochloride (Bausch & Lomb) used as a local anaesthetic. Pupils were dilated with 1% tropicamide (Bauch & Lomb) to assist with the needle positioning at the centre of the eye (Supplementary Fig. 3). On the day of injection, vectors were thawed and diluted in sterile PBS/0.001% pluronic F68 surfactant to 2.5 × 10^12^ genome copies per millilitre so that a 2 μl intravitreal injection delivered 5 × 10^9^ genome copies to each eye. Vectors were administered at random via a 5 μl Hamilton Syringe (#7803-05; Needle: 33G, 9.54 mm, point style 2; Hamilton Co.) over 30 s (Supplementary Fig. 3).

Thirty-one days after injection, mice were terminally anesthetised with Dolethal. Eyes were removed (Supplementary Fig. 4) and post-fixed in 4% paraformaldehyde (PFA) (104003100; Sigma-Aldrich) after puncturing the cornea to aid fixative penetration, or collected unfixed for protein analysis. Twenty-four hours after fixation, tissue was transferred to PBS until the time of whole-mounting/sectioning. Samples were to be excluded if the animal had a cataract or ocular bleed as a result of the intravitreal injection. These were pre-established criteria and one eye (AAV2-SYN-eGFP) was excluded from analysis. After collection of the eyes, mice were perfused with PBS followed by 4% PFA for 10 min at a flow rate of 20 ml/min. Optic nerves, optic chiasms, and brains were collected (Supplementary Fig. 4) and then placed in 4% PFA overnight, and afterwards kept in 30% sucrose in PBS and in the dark at 4 °C until the time of immunohistochemistry. Samples were blinded until after analysis.

### Retinal explant cultures and AAV-mediated transgene expression ex vivo

To assess vector transduction of human retina we used punches from donor human retina. Retina were acquired as anonymised excess donor tissue from the St. Erik Eye Hospital cornea transplant service. This study adhered to the tenets of the Declaration of Helsinki and was approved by the Swedish Ethical Review Authority, Dnr 2020-01525 (“Studier av neuronal metabolism, biomarkörer och neuroprotektion vid glaukom”). Donor details are shown in the relevant result section. Retinas were dissected in ice-cold Hank’s balanced salt solution, the vitreous removed, and the retina flattened following a modification of a previously described protocol [46]. Using a dissecting trephine, punches of retina (2 mm diameter) were taken from an arc, 4 mm nasally to the optic nerve head where the RGC density of the retina is most consistent between punches. Punches were cultured individually, RGC side up, on cell culture inserts (Millicell, 0.4 µm pore, 12 mm diameter; Merck) in 24-well plates with media only present in the well, and not within the culture insert. AAV vectors (described above) were delivered by pipetting a single 3 µl drop (2.5 × 10^12^ genome copies per ml) on to the surface of the punch. The droplet size was chosen to transduce the inner retina (mimicking an intravitreal injection) without additional media saturating the sides and the outer retina, which would transduce cells from the bottom up (i.e. outer retina infection mimicking a subretinal injection). This dose was also designed to closely mimic the effective doses in successful gene therapy trials (e.g. in [47], they administered an effective dose of 1 × 10^9^ GC per μl which is comparative to our 2.5 × 10^9^ GC per µl). We used retinal explant culture media as previously described for rodent [48] and human retina [49], consisting of Neurobasal-A media supplemented with 2 mM L-glutamate (GlutaMAX), 2% B27, 1% N2, and 1% penicillin/streptomycin (all Gibco, Merck). Each well contained 400 µl of media. Punches were maintained in culture (37 °C, 5% CO_2_) for 7 days, with a half media change every 2 days. Punches were then fixed for 30 min in 3.7% PFA added to the well in place of the media, and within the culture insert. Punches were gently lifted from the insert and placed on glass slides for immunofluorescent labelling.

For mouse retinal explants; male C57BL/6J at >10 weeks of age or male B6.BOla-*Wld*^*S*^ at >10 weeks of age were used. To generate B6.BOla-*Wld*^*S*^ mice the original *Wld*^*S*^ allele [50] was backcrossed onto a B6J background for at least 12 generations (*N* > 12). Mouse retinas followed the same protocol as donor human retinas, with the exception that whole retina was explanted immediately after euthanasia by cervical dislocation and maintained for 5 days. Mouse retinas were maintained in 6-well plates using 30 mm inserts.

### Immunohistochemistry

We opted not to include the use of anti-GFP antibodies on mouse retinal wholemounts, retinal sections, and optic chiasm sections, to limit fluorescence background and limit bias from non-specific staining. Staining for GFP protein was performed on mouse brain sections to increase the brightness of eGFP-positive axons, derived from transduced RGCs. Staining for GFP was also added to post-mortem retinal explants to boost the transgene signal.

#### Retinal wholemounts

Retinas were exposed by dissection around the circumference of the eye globe and removal of the lens. The retinas were obtained by peeling off the underlying retinal pigment epithelium and detachment of the optic nerve. The retinas were flattened and stored in PBS until immunohistochemistry procedures. Retinal wholemounts were washed in 0.5% Triton X-100 in PBS and afterwards frozen at −70 °C for 10 min to permeate the nuclear membrane and thereby improve Brn3a staining. After the freezing, the retinal wholemounts were washed again in 0.5% Triton X-100 in PBS followed by blocking in 10% normal donkey serum (D9663; Sigma-Aldrich), 2% bovine serum albumin (BSA) (A7906; Sigma-Aldrich), and 2% Triton X-100 in PBS for 1 h. The retinal wholemounts were then incubated with primary antibodies against Brn3a (C-20) (sc-31984; 1:200; Santa Cruz Biotechnology) in the above-mentioned blocking detergent for 2 h at room temperature and afterwards kept at 4 °C overnight. Afterwards, the tissue was washed in 2% Triton X-100 in PBS before washing in 0.5% Triton X-100 in PBS. The retinal wholemounts were then incubated with anti-goat IgG conjugated Alexa Fluor (AF) 647 (A21447; 1:1000; Thermo Fisher Scientific) in 2% Triton X-100 in PBS for 2 h at room temperature. The retinal wholemounts were washed in PBS and then mounted using FluorSave^TM^ reagent (345789; Calbiochem) on super frost plus slides (VWR).

#### Retinal sections

Whole eyes were cryo-preserved by immersion in 30% sucrose overnight at 4 °C, followed by embedding in optimal cutting temperature compound (OCT) (Sakura Finetek). Eyes were then frozen on dry ice and 13 μm sections collected through the dorsal–ventral/superior–inferior axis of the retina onto super frost plus slides using a OTF5000 cryostat (Bright instruments). Sections were washed in PBS and blocked in 5% normal goat serum (NGS) (G2023; Sigma-Aldrich), 2% BSA, and 0.3% Triton X-100 in PBS for 60 min at room temperature. Sections were then incubated in primary antibodies against RBPMS (1832; 1:500; PhosphoSolutions), Prox1 (925202; 1:500; BioLegend), Calretinin (ab702; 1:500; Abcam), Calbindin (ab11426; 1:500; Abcam), PKC-α (sc-8393; 1:500; Santa Cruz Biotechnology) or Vimentin (ab5733; 1:500; Millipore). Afterwards, the tissue was washed in PBS then incubated with secondary antibodies anti-guinea pig AF 555 (A21435; 1:1000; Thermo Fisher Scientific), anti-chicken AF 568 (A11041; 1:1000; Thermo Fisher Scientific), anti-rabbit AF 647 (A32733; 1:1000; Thermo Fisher Scientific) or anti-mouse AF 555 (A21424; 1:1000; Thermo Fisher Scientific) with DAPI (D1306; 1:8000; Thermo Fisher Scientific) in the above-mentioned blocking detergent for 2 h at room temperature. The retinal sections were washed in PBS and then mounted using FluorSave^TM^ reagent.

#### Optic chiasm sections

The collected optic chiasms were carefully positioned flat on independently, freshly sectioned blocks of OCT. The chiasms were frozen and cut into 30 μm thick ventral sections using a Leica CM3050 S cryostat and mounted on super frost plus slides. Each section was examined under direct fluorescence microscopy until the complete chiasm was visible. The optimal slide for each chiasm was washed in PBS before incubation with DAPI (1:8000) in PBS for 20 min in the dark at room temperature. Afterwards, the tissue was washed in PBS and mounted using FluorSave^TM^ reagent.

#### Brain sections

Mice brains were embedded in silicone moulds containing OCT, frozen and cut into 30 μm thick sagittal- and coronal sections using a Leica CM3050 S cryostat and mounted on super frost plus slides. Slides were washed in PBS and afterwards permeabilized and blocked by incubation in 10% NGS and 0.3% Triton X-100 in PBS for 2 h. The brain sections were then incubated with primary antibody against GFP (ab290; 1:800; Abcam) in the above-mentioned blocking detergent in the dark at 4 °C overnight. After incubation, the tissue was washed in PBS before incubation with anti-rabbit AF 488 (A11008; 1:1000; Thermo Fisher Scientific) and DAPI (1:8000) in blocking solution for 2 h at room temperature. The brain sections were washed in PBS and mounted using FluorSave^TM^ reagent.

#### Post-mortem retinal explants

Cultured human punches and mouse whole retina followed the same labelling protocol. Following fixation, retina were transferred to glass slides and isolated using a hydrophobic barrier marker. Retinas were permeabilized in 0.5% Triton X in PBS for 1 h, blocked using 2% BSA in PBS for 1 h, and then incubated overnight at 4 °C with primary antibodies against RBPMS (NBP2-20112, 1.3 µg/ml working concentration, Novusbio) and GFP (ab13970, 20 µg/ml working concentration. Abcam). We opted to use anti-GFP in these retinas to counteract the autofluorescence of the tissue following culture. Retinas were washed five times for 5 min in PBS and incubated with secondary antibodies anti-rabbit AF 568 (A11011, 4 µg/ml working concentration, Invitrogen) and anti-chicken AF 488 (A11039, 4 µg/ml working concentration, Invitrogen) for 4 h at room temperature. Retinas were then washed in PBS five times for 5 min, nuclear stained using DAPI (1 μg/ml) and mounted using Fluoromount-G (Invitrogen). Slides were cover-slipped and sealed.

### Microscopy

HEK293T cells were imaged live using a FLoid^TM^ Cell Imaging Station (4471136; Thermo Fisher Scientific) using a ×20 objective. Representative overview images of retinal wholemounts, retinal eye cups, and brain sections, were all taken using a tile-scanning epifluorescence microscope (Leica, DMi8) with a ×20 objective. High magnification images of retinal wholemounts were taken using a confocal microscope (Leica, TCS SPE, DMI4000B) with a ×40-oil objective. Twelve images (three images per retinal quadrant to provide a global, unbiased, overview of the inner retina) were captured per retinal wholemount to determine the AAV expression per eye. The RGC layer was exclusively imaged with the confocal microscope to remove possible co-localisation discrepancies from transduced deeper layer cells to improve the accuracy of transgene expression quantifications. Retinal cross-sections were imaged using an epifluorescence microscope (Leica, DM6000) with a ×20 objective. Horizontal sections containing optic nerves, optic chiasms, and optic tracts, were imaged using a tile-scanning epifluorescence microscope (Leica, DMi8) with a ×20 objective. High magnification pictures of the superior colliculus and the lateral geniculate nucleus (LGN) were captured using a confocal microscope (Leica, TCS SPE, DMI4000B) with a ×40-oil objective. Retinal explants were imaged using a confocal microscope (Zeiss, LSM800-Airy). Image settings were kept constant between samples for both human or mouse retinal tissue. For human punches, the centre of the punch was imaged at ×20 magnification (0.7X optical zoom), giving a field of view of 450 × 450 µm. For mouse retinas, six images (450 × 450 µm) were acquired equidistant to the optic nerve head, according to previous methods [48].

### Quantification of histological samples

The quantification of the expression efficiency of viral vectors and the transgene expression levels in retinal wholemounts was analysed using the ImageJ plugins “RGC Transduction” and “RGC batch” that are part of “Simple RGC” (version 1.1.0) [51]. This software identified co-localisation between Brn3a-postive cells (RGCs) and eGFP-positive cells (transduced cells) to specifically identify transduced RGCs. It next determined: (1) the eGFP intensity for each individual transduced RGC; (2) the average eGFP intensity in transduced RGCs per image; and (3) the viral expression efficiency as a percentage by dividing the number of transduced RGCs by the total number of RGC. The following image processing parameters were set for the automated quantification of transduced RGCs and the eGFP fluorescence intensity: cell diameter (pixels): 20–60; local threshold radius: 60; Gaussian blur sigma: 3. Retinal sections were manually processed for co-localisation by switching channels on and off using Leica LAX software. In total, 48 images, each spanning a 700 μm linear section of retina, were assessed per promoter and used to create the tropism pie charts. These images were collected from at least eight separate ocular sections taken none sequentially over multiple slides and at multiple positions from the optic nerve head to the peripheral retina to give a global overview of transduction. For retinal explants, RBPMS+, GFP+, and co-labelled cells were counted using the cell counter plugin in Fiji [52], where the image was cropped to the central 200 × 200 µm.

### Western blot and quantification

Four weeks after AAV injection, eyes were removed, and retinas carefully dissected from the eye cup and snap frozen on dry ice. Retinas were lysed using 125 µl Lysis-M reagent containing complete Mini Protease Inhibitor (Roche) and soluble cell extracts quantified via a bicinchoninic acid (BCA) protein assay (Thermo Fisher Scientific). In total, 5 µg of protein was loaded into a 4–12% Bis-Tris gel (NuPAGE Novex, Thermo Fisher Scientific) and PVDF membrane probed for GFP (ab290; 1:10,000; Abcam) and β-actin (4967; 1:1000; Cell Signalling). Signal detection was using ECL Prime (GE Healthcare) and an Alliance Western blot imaging system (UVItec Ltd). Blots were repeated on two occasions to validate results and band area quantified in ImageJ using the “plot profile” feature.

### Statistical analysis

Statistical analysis was performed by using Graphpad Prism 9.00 for Mac OS X. All statistical tests and parameters are mentioned in the figure legends. The graphs represent the mean for each condition together with the individual data points for each animal. For fluorescence intensity measurements, data are also shown for each transduced RGCs to demonstrate spread and uniformity of expression. The correlation between the number of total RGCs and the number of transduced RGCs in post-mortem retinal explants was performed using nonparametric Spearman correlation analysis as the data did not pass the Shapiro–Wilks normality test.

## Results

### Effect of Kozak consensus sequence on retinal transgene expression

The inclusion of a Kozak sequence has been shown to enhance protein translation [53–57]. To empirically test this, the effect of the Kozak consensus sequence (GCCACCATGG) on AAV2-mediated expression of eGFP in the retina was evaluated. We compared the construct AAV2-CBA-eGFP without a Kozak, to AAV2-CBA-eGFP containing a Kozak, and assessed the strength of eGFP protein expression by Western blotting. Both AAV vectors were delivered to the retina via intravitreal injection and retinal lysates were harvested after 4 weeks.

**Fig. 2:**
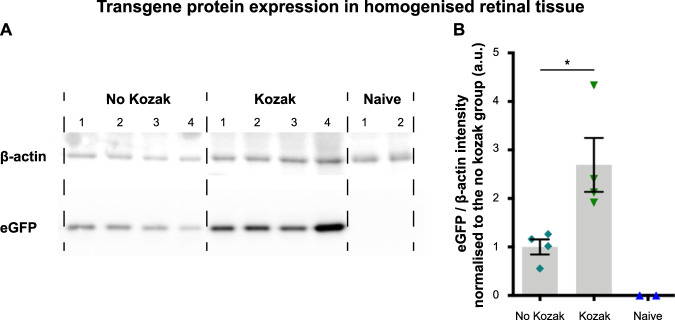
The effect of the Kozak consensus sequence on eGFP protein expression in the retina. **A** eGFP (27 kDa) and loading control β-actin (45 kDa) were assessed in retinal lysates 4 weeks after intravitreal injection of 5 × 10^9^ genome copies per eye of AAV2-CBA-eGFP excluding the Kozak sequence (*n* = 4) or AAV2-CBA-eGFP including the Kozak sequences (*n* = 4). Eyes that were not injected were processed identically as naïve controls (*n* = 2). **B** Quantification of the mean eGFP intensity per transduced retina is shown, corrected for total protein loaded per sample (df = 6, *t* = 2.93, *p* < 0.05, Student’s *t* test for No Kozak vs. Kozak). The grey bars depict the mean ± SEM. Each individual data point represents one retina. Images were taken from the same Western blot; different exposure times were needed to visualise β-actin and eGFP proteins, respectively. *p < 0.05.

eGFP protein was expressed by both AAV2 vectors and it was non-existent in retinal lysates of naïve eyes (Fig. 2). Importantly, the inclusion of a Kozak sequence generated ~2.5-fold more intense eGFP signal (2.7 ± 0.6 a.u) than the construct lacking the Kozak (1.0 ± 0.2 a.u) (Fig. 2). It was concluded that the addition of the Kozak consensus sequence enhances expression of the eGFP transgene and was therefore included in further AAV vectors in this study.

### Expression in the inner retina following intravitreal injection of AAV2 vectors

**Fig. 3:**
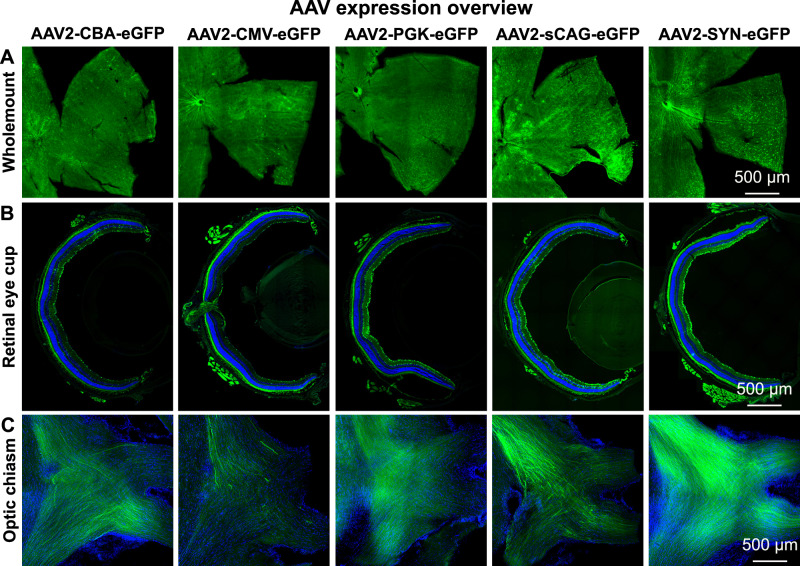
Expression overview in mice that received intravitreal injections of AAV2-CBA-eGFP, AAV2-CMV-eGFP, AAV2-PGK-eGFP, AAV2-sCAG-eGFP, or AAV2-SYN-eGFP. eGFP (green) is shown in the retinal wholemount quadrants (**A**), retinal eye cups (**B**) and optic chiasms (**C**); DAPI staining (blue) was performed on the retinal eye cup and the optic chiasm sections to improve histological visualisation. Autofluorescence emission from extraocular muscle and orbital fat is visible in the sections of retinal eye cups. All overview images were taken with a tile-scanning epifluorescence microscope with identical microscope settings between the experimental groups per retinal tissue. eGFP is shown throughout without amplification using a GFP antibody.

In total, 5 × 10^9^ genome copies of AAV2-CBA-eGFP, AAV2-CMV-eGFP, AAV2-PGK-eGFP, AAV2-sCAG-eGFP, and AAV2-SYN-eGFP were intravitreally injected into eyes of adult mice and histology on various components of the visual system was prepared after 4 weeks. Representative overview images from each AAV vector demonstrated successful expression in vivo (Fig. 3). Further analyses were then carried out to determine the strength of transgene expression and the cellular expression profile of each promoter in the tissues of these mice.

### Efficiency of AAV2 and five promoters to express eGFP in retinal ganglion cells

**Fig. 4:**
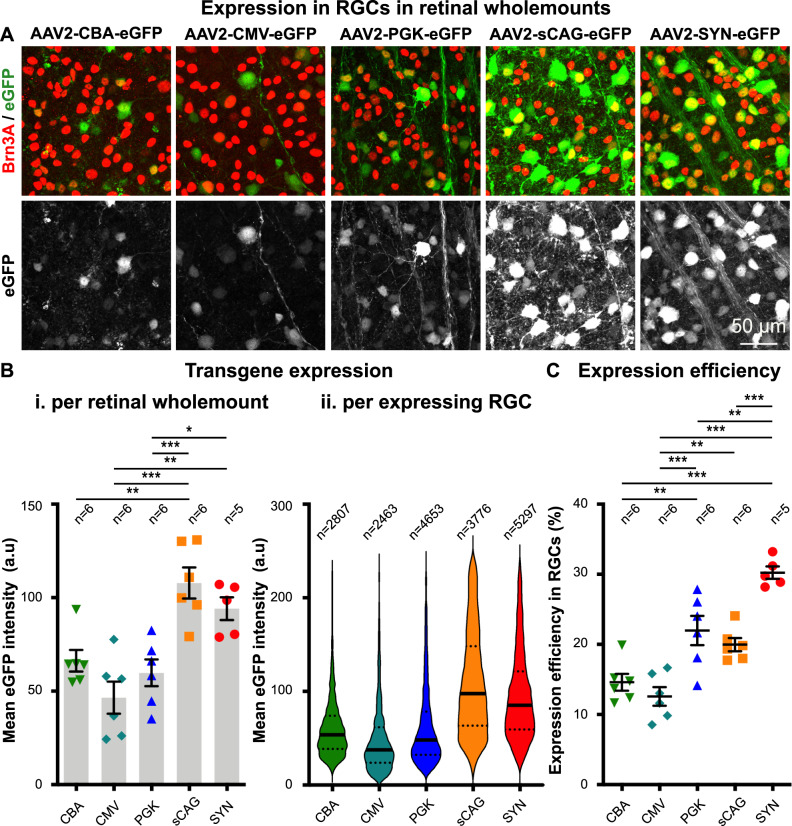
The expression efficiency and mean eGFP intensity in retinal ganglion cells after intravitreal injection of AAV2 and five promoters in mice. **A** Brn3A staining (red) and eGFP expression (green in upper panels and grey in lower panels) in retinal wholemounts of mice were intravitreal injected with AAV2-CBA-eGFP, AAV2-CMV-eGFP, AAV2-PGK-eGFP, AAV2-sCAG-eGFP, and AAV2-SYN-eGFP. **B** Quantification of the mean eGFP intensity in transduced RGCs shown as average for each retinal wholemount (i) (df = 4(24), *F* = 11.9, *p* < 0.001, ANOVA with Tukey’s multiple comparison test) and as a visualisation of the eGFP intensity for each expressing RGC (ii). **C** Quantification of the expression efficiency of AAV2 with promoters for RGCs in retinal wholemounts (df = 4(24), *F* = 23.3, *p* < 0.001, ANOVA with Tukey’s multiple comparison test). **B**i, **C** depict the mean ± SEM in which each individual data point represents the mean value of twelve images within one retinal wholemount. **B**ii shows a violin plot containing the values of all RGCs expressing the eGFP transgene in which the line represents the median and the dotted patterns the quadrants. Images were taken using a confocal microscope with identical microscope settings between the experimental groups. eGFP is quantified and shown throughout without amplification using a GFP antibody. *p < 0.05; **p < 0.01; ***p < 0.001.

A key aim of this study was to identify which promoters could transduce the greatest number of RGCs, as such promoters would be suitable for researchers wishing to target or regenerate RGCs. Four weeks after intravitreal AAV2 delivery, transduced retinal wholemounts were imaged (Figs. 3A and 4A) and the strength of transgene expression within RGCs was quantified using automated image analysis [51].

Both sCAG and SYN promoters had the highest expression levels of eGFP in RGCs (108 ± 8 and 94 ± 6 a.u., respectively), ~2-fold greater than the other promoters (Fig. 4A, B). eGFP intensity was relatively similar between the CBA (65 ± 6 a.u.), CMV (46 ± 9 a.u.), and PGK (60 ± 7 a.u.) promoters (Fig. 4A, B). The SYN promoter transduced the highest proportion of RGCs (30 ± 2% per retinal wholemount), followed by PGK and sCAG (22 ± 2 and 20 ± 1% per retinal wholemount, respectively) (Fig. 4A, C). Lower RGC expression was noted in the CBA and CMV groups by comparison (15 ± 1 and 13 ± 1% per retinal wholemount, respectively) (Fig. 4A, C). Taken together, the sCAG and SYN promoters provide strong transgene expression with the SYN promoter targeting the greatest RGC number and specificity. The transduced retinal wholemounts also showed that the investigated promoters do not solely target RGCs (e.g. eGFP in Brn3a-negative cells) and therefore the cellular expression profile was assessed in retinal cross-sections.

### The expression profile of AAV2 and five promoters

**Fig. 5:**
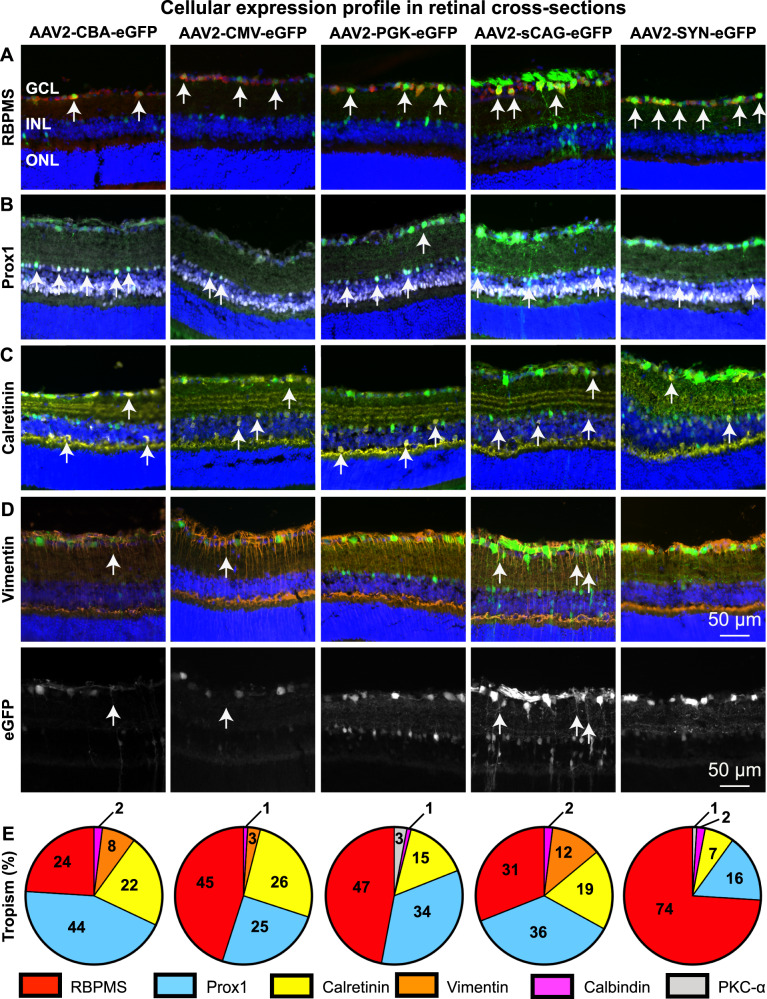
The cellular expression profile in the mouse retina after intravitreal injection of AAV2 and five promoters. eGFP expression (green) and DAPI staining (blue) together with RBPMS (**A**, red), Prox1 (**B**, cyan), Calretinin (**C**, yellow), and Vimentin (**D**, orange) staining in retinal cross-sections of mice that were intravitreal injected with AAV2-CBA-eGFP, AAV2-CMV-eGFP, AAV2-PGK-eGFP, AAV2-sCAG-eGFP, and AAV2-SYN-eGFP. **E** Quantification of the cellular tropism by each promoter expressed as a percentage of all transduced cells assessed. Two eyes, each from a different mouse, were analysed for each promoter. Images were taken using an epifluorescent microscope with identical microscope settings between the experimental groups per investigated cell-type. eGFP is shown throughout without amplification using a GFP antibody. GCL ganglion cell layer, INL inner nuclear layer, ONL outer nuclear layer.

Retinal cross-sections were examined to visualise and calculate the expression pattern within various layers of the inner retina for each promoter (Figs. 3B and 5 and Supplementary Fig. 5). Immunohistochemical markers covering most inner retinal neurons were selected, in addition to vimentin to assess Müller glia and astrocyte expression. RNA-Binding Protein with Multiple Splicing (RBPMS) is a pan-RGC marker expressing exclusively in the RGC layer whilst Prospero-related homeobox 1 (Prox1) is a specific marker for AII amacrine cells. Calretinin is a calcium binding protein found in both RGCs and amacrine cells and Calbindin primarily labels amacrine and horizontal cells. Protein kinase C-alpha (PKC-α) was chosen as a marker for rod bipolar cells. As expected from intravitreally injection of AAV2 vectors, we found no evidence of photoreceptor transduction (Fig. 3B) and the transduction of horizontal and bipolar cells was poor (Fig. 5E and Supplementary Fig. 5).

The CBA promoter had moderate transgene expression and an overall low expression efficacy throughout the retina (Fig. 4). This ubiquitous promoter did not preferentially express in any given cell type, as eGFP was observed in all investigated neuronal types and glia (44% Prox1+, 24% RBPMS+, 22% Calretinin+, 8% Vimentin+, 2% Calbindin+ of total transduced cells; Fig. 5, column 1). Likewise, the CMV promoter showed a ubiquitous expression pattern (45% RBPMS+, 25% Prox1+, 26% Calretinin+, 3% Vimentin+, 1% Calbindin+; Fig. 5, column 2). The PGK promoter showed efficient expression in RGCs, supporting the wholemount data (Fig. 4A, C), and expression was also observed in a high proportion of amacrine cells, but Müller glial cells were not transduced (47% RBPMS, 34% Prox1+, 15% Calretinin+, 3% PKC-α+, 1% Calbindin+; Fig. 5, column 3). The sCAG promoter gave strong transgene expression (Fig. 4A, B) and retinal cross-sections highlighted a broad cellular tropism with this promoter for inner retinal neurons and Müller glial cells (36% Prox1+, 31% RBPMS+, 19% Calretinin+, 12% Vimentin+, 2% Calbindin+; Fig. 5, column 4). The SYN promoter drove strong transgene expression in numerous RGCs (Fig. 4), and expression was also found to be mostly localised to the ganglion cell layer. Among the investigated promoters, SYN was the most selective for RGCs with only a small proportion of amacrine cells weakly transduced. No transduced glial cells were visible (74% RBPMS+, 13% Prox1+, 7% Calretinin+, 2% Calbindin, 1% PKC-α+; Fig. 5, column 5).

### Detection of eGFP-positive axons from transduced RGC along the visual pathway

**Fig. 6:**
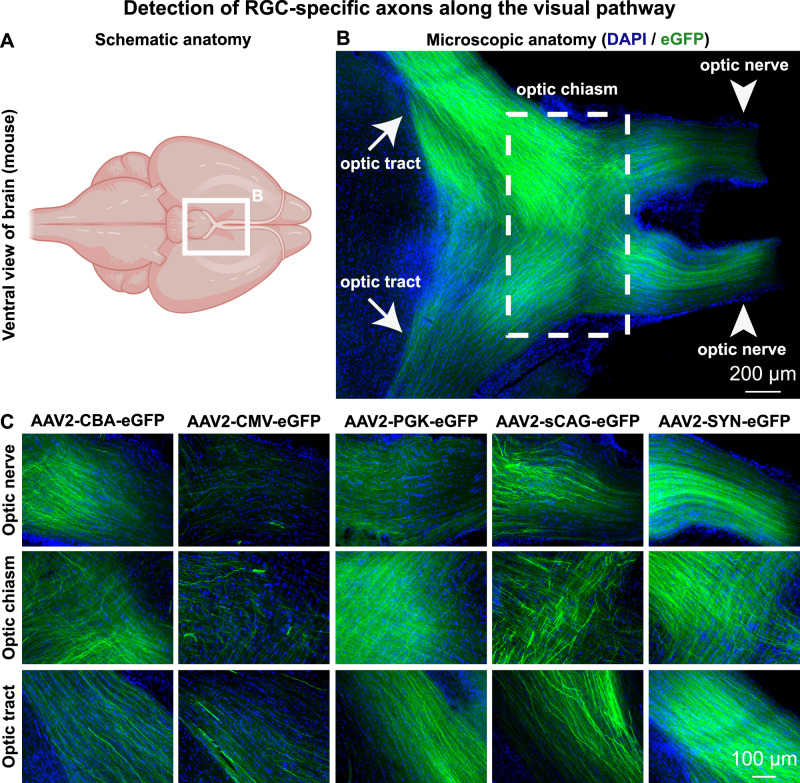
eGFP-positive axons of transduced mouse retinal ganglion cells are detected at the optic nerve, optic chiasm and optic tract. **A** Schematic representation of a ventral view on the mouse brain, in which the white square illustrates the location of the optic chiasm and related structures. **B** Microscopic overview of the optic chiasm from a mouse that had bilateral AAV2-SYN-eGFP intravitreal injections. This image is modified from the panel containing AAV2-SYN-eGFP in Fig. 3C. **C** eGFP-positive fibres were found at the optic nerve, optic chiasm and optic tract in all investigated experimental groups. All images were taken using a tile-scanning epifluorescence microscope with exposure settings selected to maximise axon visualisation for each promoter. Horizontal sections were stained for DAPI, eGFP is shown throughout without amplification using a GFP antibody. Schematic was created with BioRender.com.

**Fig. 7:**
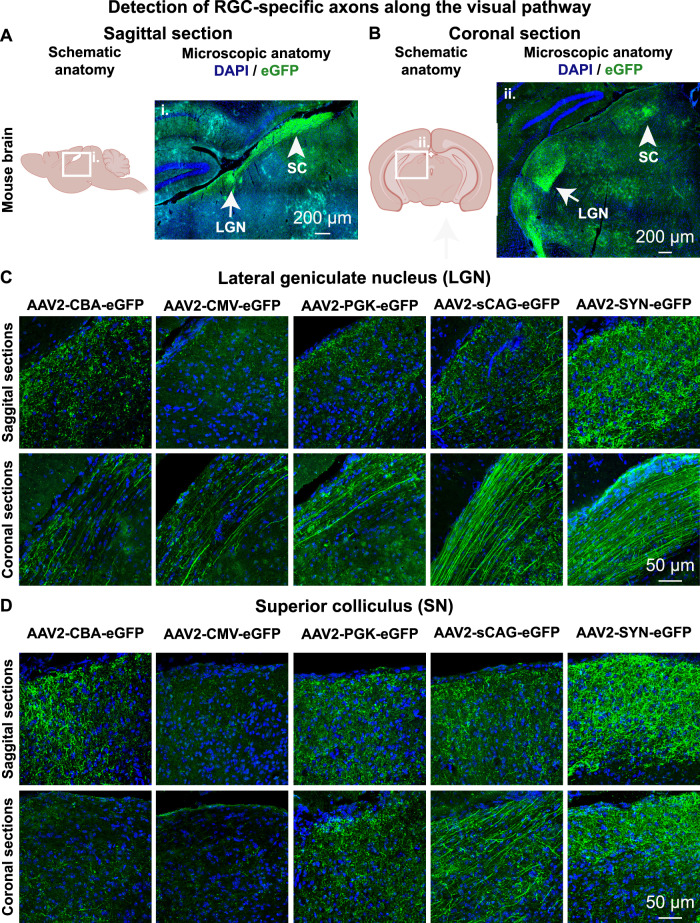
eGFP-positive axons of transduced mouse retinal ganglion cells are detected at the LGN of the thalamus and the SC. **A**, **B** Sagittal and coronal sections of the mouse brain containing the LGN and SC in one hemisphere shown as a schematic and as a microscopic overview image from a mouse that had bilateral AAV2-SYN-eGFP intravitreal injections. All investigated experimental groups had eGFP-positive fibres projecting to the LGN (**C**) and SN (**D**). The overview image was taken with a tile-scanning epifluorescence microscope, whilst magnified images were taken using a confocal microscope with identical settings between experimental groups. All brain sections were stained for DAPI and eGFP. Schematic was created with BioRender.com. LGN lateral geniculate nucleus, SC superior colliculus.

Examining eGFP distribution from AAV2 vectors with promoters throughout the visual pathway demonstrated it was possible to visualise eGFP-positive axons in the optic nerve, the optic chiasm, and the optic tract (Figs. 3C and 6). These fluorescent axons, whose fluorescence was enhanced by immunohistochemistry for GFP, are derived specifically from transduced RGC and terminated in the LGN of the thalamus and the superior colliculus as shown in sagittal- and coronal sections of the mouse brain (Fig. 7). Transgene expression strength and specificity matched that seen in retinal wholemounts and cross-sections, with the SYN promoter providing evidence of strong, uniform expression in the largest number of axons (Figs. 6 and 7). In summary, intravitreal injection of AAV2 with the SYN promoter was the best to target RGCs, and expression of the eGFP transgene was seen along the visual pathway in the brain, whereas expression by the other investigated promoters was also observed in the brain, it was less efficient and they had a higher degree of expression of non-RGCs.

### Expression of AAV2 and five promoters in post-mortem human retinal explants

**Fig. 8:**
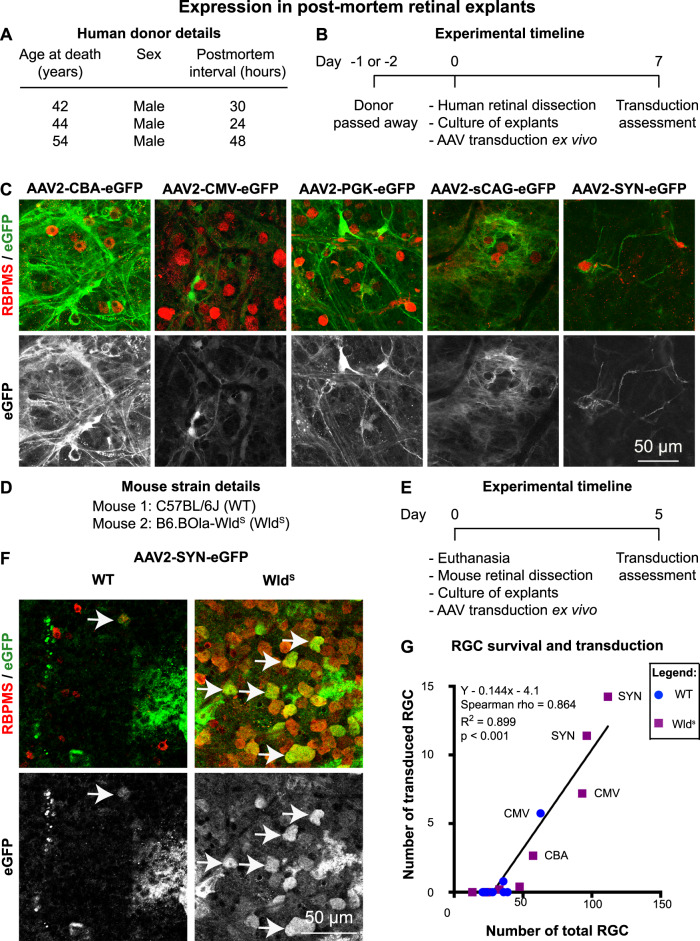
Expression of AAV2-CBA-eGFP, AAV2-CMV-eGFP, AAV2-PGK-eGFP, AAV2-sCAG-eGFP, and AAV2-SYN-eGFP in post-mortem human retinal explants. **A** Table highlighting human donor details. **B** Time course of the post-mortem human retinal explant preparation, viral transduction and expression assessment. **C** RBPMS staining (red) and eGFP staining (green in upper panels and grey in lower panels) in post-mortem human retinal explants that were transduced with AAV2-CBA-eGFP, AAV2-CMV-eGFP, AAV2-PGK-eGFP, AAV2-sCAG-eGFP, and AAV2-SYN-eGFP ex vivo. Images were taken using a confocal microscope with identical microscope settings between the experimental groups. Human explants were stained for eGFP. **D** Mouse strains used for retinal explants. **E** Time course of the post-mortem mouse retinal explant preparation, viral transduction and expression assessment. **F** RBPMS staining (red) and eGFP staining (green in upper panels and grey in lower panels) in post-mortem retinal explants of C57BL/6J and B6.BOla-*Wld*^*S*^ mice that were transduced with AAV2-SYN-eGFP ex vivo. Mouse explants were stained for eGFP. **G** Comparison between the number of total RGCs and the number of transduced RGCs in post-mortem mouse retinal explants transduced with AAV2-CBA-eGFP, AAV2-CMV-eGFP, AAV2-PGK-eGFP, AAV2-sCAG-eGFP, and AAV2-SYN-eGFP ex vivo [*Y* = 0.176x − 5.5, Spearman’s rho = 0.864, *R*^2^ = 0.899, *p* < 0.001, nonparametric Spearman correlation]. *N* = 7 for C57BL/6J (WT) and *N* = 9 for B6.BOla-*Wld*^*S*^.

We next assessed whether these vectors were able to transduce human retina ex vivo (Fig. 8A). Each AAV2 vector was added directly to the inner retinal surface of cultured retinal punches taken from whole donor retina 24–48 h post-mortem (Fig. 8B). At 24–48 h post-mortem, RGC degenerative processes (i.e. Wallerian degeneration and apoptosis) have initiated. The extent of RGC death during the following 7-day culture period varied per donor tissue, but was comparable between punches exposed to the different AAV vectors (Supplementary Fig. 6). All promoters (CBA, CMV, PGK, sCAG, SYN) were able to drive some eGFP expression in the human retina after 7 days ex vivo (Fig. 8C). High expression was observed at the retina punch border (Supplementary Fig. 7) and it was predominantly restricted to glia, particularly astrocytes and Müller glia end feet, with limited neuronal expression (likely amacrine cells) (Fig. 8C), probably due to low viability of neurons in post-mortem tissue and a thick inner limiting membrane that is present in humans (this is absent to very thin in rodent retina).

To determine whether the lack of transgene expression in RGCs in post-mortem human tissue was related to the viability of RGCs, we investigated post-mortem retinal explants from C57BL/6J and B6.BOla-*Wld*^*S*^ mice (Fig. 8D). B6.BOla-*Wld*^*S*^ mice were chosen as they carry the *Wld*^*S*^ allele which dramatically reduces Wallerian degeneration and as such are partially resilient to axotomy-induced neuronal death [50] that is initiated by explanting a retina (axotomy of the RGC). Mouse retinal explants were transduced with AAV2 harbouring promoters on the day of death, and cultured for 5 days in the same manner as human retinal punches (Fig. 8E). Spearman correlation analysis demonstrated a strong positive correlation between RGC survival and expression ex vivo (Fig. 8F, G, Spearman’s rank correlation coefficient of 0.864). As expected, transduction efficiency was higher in non-degenerative systems (i.e. mice carrying the *Wld*^*S*^ allele) even when the RGCs are axotomized.

## Discussion

This study examined the efficiency and selectivity of five promoters for gene expression in the mouse inner retina. The size of the promoter is an important consideration when delivering large transgenes [58], bi-cistronic proteins or complex cargo [59–61] using AAVs. For this reason, we selected promoters under 868 bp in length, therefore giving a theoretical cargo of ~4000 bp. The selected promoters are also commonly used for gene transfer in the nervous system, making them ideal candidates for screening and developmental purposes.

The eyes investigated in this study were injected with 5 × 10^9^ genome copies per eye, to titre-match the viral vectors, and to optimise the detection of potential subtle differences between experimental groups. The AAV vectors were administrated via intravitreal injection to achieve optimal targeting of the ganglion cell layer and expectedly, deeper retinal layer transduction was restricted. We examined promoter driven expression in retinal wholemounts and retinal cross-sections 4 weeks after AAV2 delivery, a time-point that provides optimal expression [10, 11, 29, 33, 62] and is therefore relevant for many AAV-mediated gene transfer studies in the retina. It should be noted that injecting a higher titre vector, adding regulatory units, or using modified AAV2 capsids could enhance the transduction efficiency further. These factors were not examined in this study in order to achieve an experimental design that is optimal to directly compare the activity of relatively short promoters and using a minimal amount of space within the AAV2 genome.

It should also be noted that other researchers have tested a range of promoters to target different retinal cell populations following intravitreal delivery (Table 1). These promoters should also be considered, if cargo space allows, when designing AAV vectors to target the retina (including cells other than RGCs). As the field of gene therapy is increasingly tilting towards the use of cell- and tissue-specific promoters, it is also important that DNA sequences of promoter regions are publicly available.

**Table 1:** Other promoters investigated in the retina after intravitreal injection of AAV.

Promoter	Size	Reference
Neurofilament light polypeptide	2693 bp	[25]
Neurofilament heavy polypeptide	2251 and 2501 bp^a^	[26, 27]
Doublecortin	2359 bp	[29]
Phosphodiesterase 6H	2005 bp	[30]
Purkinje cell protein 2	1652 bp	[31]
Gamma-synuclein promoter	1450 and 953 bp^a^	[32, 33]
Interphotoreceptor-binding protein	1300 bp	[34]
Short promoter variant of glial fibrillary acidic protein	694 bp	[136]
Monocyte chemo attractant protein-1	560 bp	[36]
Short promoter variant of mouse cone arrestin	521 bp	[37]
Short promoter variant of human neurofilament heavy polypeptide	199 bp	[27]

*bp* base pair.

^a^Indicates that the size is shown for the mouse and human promoter, respectively.

### Transgene expression in HEK293T cells after plasmid transfection

AAV vector plasmids were pre-validated prior to AAV production and subsequent animal studies by DNA sequencing, the presence of two ITR sequences, and transgene expression in transfected HEK293T cells. Expression was investigated in this human cell line because it is commonly used to produce AAVs [63, 64]. We found that all promoters initiate transgene expression in cultured HEK293T cells, including weak expression by SYN. There are a few possible explanations why this neuronal-specific promoter still initiated weak expression in these cells derived from human-embryonic kidney: (1) the high copy-number of the transgene due to the plasmid DNA transfection may be responsible for leaky expression; (2) ITR sequences in the plasmid DNA have weak promoter activity themselves [65, 66] and may cause expression in these non-neuronal cells; (3) there are reports that the SYN promoter can initiate expression in non-neuronal cells such as hepatocytes after high-dose and systemic administration [67–69]. In contrast to observations in HEK293T cells, we’ve previously demonstrated that the SYN promoter does initiate strong transgene expression in cultured CNS neurons [45].

### Kozak consensus sequence enhances transgene expression of AAV vectors

The Kozak consensus sequence aids ribosomes in identifying the start codon on transcribed mRNA to begin protein translation [53–57]. We investigated whether the insertion of the most optimal, vertebrate Kozak sequence in AAV2 vectors enhanced expression of the eGFP transgene in the mouse retina. The examined Kozak consensus sequence had the following composition: (1) the sequence 5’ GCCACC 3’ directly upstream of the start codon; (2) the start codon 5’ ATG 3’; (3) the first nucleotide base of the second codon of the transgene which is guanine 5’ G 3’. We found that the Kozak consensus sequence increased the eGFP transgene signal by ~2.5-fold in an AAV2 vector harbouring the CBA promoter. An increase in transgene expression is consistent with the hallmark studies by Marilyn Kozak that examined protein translation using non-AAV vectors [53–57]. The inclusion of a Kozak sequence during AAV vector design is therefore beneficial for enhancing eGFP expression at the cost of a minimal increase in nucleotide base pairs. It will be important to determine whether the Kozak consensus sequence also enhances expression of other transgene, or promoter combinations, that may be in the interest of experimenters. The Kozak consensus sequence is smaller in size than other factors promoting higher expression of transgenes, such as chimeric introns [70–72] and posttranscriptional regulatory elements [73–75], and can therefore be preferred if the AAV genome size is a limitation. Recently, a retina-derived Kozak sequence has been identified that may further enhance transgene expression in the retina [76], but its inclusion remains to be investigated using AAVs in vivo.

### Comparison of five promoters for transgene expression

AAV2 cell entry is primarily achieved by binding heparin sulfate proteoglycans [77–81] and interacting with co-receptors on the cell surface such as the adeno-associated virus receptor [82, 83], the 37/67-kilodalton laminin receptor [84], αVβ5 integrin [85], hepatocyte growth factor receptor [86], and fibroblast growth factor receptor 1 [87]. Despite the broad cellular tropism of this serotype, our study confirms that the promoter determines the degree of transgene expression once the viral genome enters the cell (Table 2).

We identified that AAV2 carrying CBA- and CMV promoters had ubiquitous expression in retinal cell types, moderate transgene expression and a relatively low expression efficacy compared to the other investigated promoters. The broad expression pattern by CMV in the retina has been observed before [13, 26, 27, 32, 88, 89]. Furthermore, our finding that RGCs only make up 20–25% of the transduced cells is also consistent with a previous study that quantified the cellular tropism using this AAV serotype and promoter [32]. The expression in multiple retinal cell types, including RGCs and amacrine cells, after intravitreal injection of AAV2-CBA is also consistent with previous reports [11, 90]. Furthermore, similar ubiquitous expression patterns by the CBA- [91] and CMV promoters [45, 74, 91–94] have been observed in the brain. The weaker transgene expression in RGCs by AAV2-CMV compared to AAV2-SYN has been reported in adult rat retina [95]. Despite reports that the CMV promoter is vulnerable to epigenetic silencing [96–99], we and others have found transgene expression using this promoter in the retina of adult mice at 4 weeks and beyond after AAV delivery [10, 12, 26, 27, 32, 89, 100]. The CMV promoter is also incorporated into gene therapy treatments for Leber Hereditary Optic Neuropathy (GenSight) and Age-related Macular Degeneration (Adverum) which are progressing through clinical trials [101–103] with limited evidence of silencing. Of note, the CMV promoter, along with a β-globin intron in some studies, is commonly used to deliver *Cre* recombinase and regeneration-associated genes in the retina of transgenic mice to promote optic nerve regeneration [104–109]. The CBA promoter has also been used to express regeneration-associated genes to promote RGC survival and optic nerve regeneration in mice [58].

The PGK promoter had moderate transgene expression, a good expression efficacy, and was preferentially expressed in RGCs and AII amacrine cells. The superior RGC expression by PGK over the CMV promoter is consistent with a study that examined AAV2-mediated transduction after optic nerve injury in mouse [14]. We demonstrated that up to 50% of the transduced cells are RGCs after intravitreal injection of AAV2-PGK which is quantitatively similar to previously published data [33]. We also observed that the PGK promoter did not initiate transgene activation in Müller glia cells in the uninjured retina. The predominant neuronal expression by AAVs carrying the PGK promoter is also observed in naive brain [45, 110, 111]. However, it has been reported that the PGK promoter does activate transgenes in Müller glia cells when the retina is injured [14]. The transgene expression driven by the PGK promoter had a similar strength to the CBA- and CMV promoters in this study. In the brain and corticospinal tract, we previously found that the PGK promoter with an additional ~400 bp-long β-globin intron had transgene expression similar to the SYN promoter [45]. Therefore, the moderate expression observed in this study could be due to the removal of this intronic sequence, in line with reports about their capability to enhance transgene expression [70–72]. However, a direct comparison between a viral vector containing a β-globin intron and one without was not performed because we were interested in the stripped back AAV vector only to obtain a maximum size capacity for the insertion of large and complex transgenes. To our knowledge, the PGK promoter has not been applied to deliver regeneration-associated genes for mouse optic nerve regeneration experiments yet.

CAG is a hybrid synthetic promoter consisting of three components: the CMV early enhancer element, parts of the chicken β-actin gene, and a β-globin intron. The full-length 1800 bp-long CAG promoter is extensively used in retinal research [29, 88, 112–116] and has become a common option when translating therapies to patients with ocular disorders [20]. Many shorter variants of this promoter are available to increase the cargo capacity for transgenes in AAVs. This study examined a 868 bp variant of this promoter containing a short/minimal β-globin intron. We demonstrated that AAV2 including this short promoter had strong transgene expression and a broad cellular tropism in the retina. The observed cellular expression profile is similar to the CBA promoter, which was expected as the promoter sequences are alike, but the sCAG promoter additional components may contribute to the stronger expression observed in the retina. The strong and broad cellular tropism of AAV2 with the short variant promoter is in line with previous studies that examined the full-length CAG in the mouse retina [29, 112, 113], although a direct comparison between the short and full-length CAG promoters has not been completed. Consistent with the cellular tropism observed in our study, Wang et al. showed that ~30% of the transduced cells in the retina were RGCs using AAV2 with a 985 bp variant of the CAG promoter [33]. The CAG promoter has been used to deliver *Cre* recombinase and various regeneration-associated genes in the retina for optic nerve regeneration experiments [117–120], including the short promoter variant investigated in this study [121].

AAV2 harbouring the SYN promoter had strongest transgene expression and the highest RGC viral expression efficacy in the mouse retina compared to the other promoters. The SYN promoter is often described as a neuron-selective promoter [45, 95, 122–125] and we consistently found transgene expression in inner retinal neurons throughout all animals. The SYN promoter can transduce multiple classes of neurons in the retina, although we noted strongest expression within RGCs when the viral vector was administrated intravitreally. The preferential expression in the ganglion cell- and inner nuclear layer of the retina by AAV2-SYN is consistent with previous reports [33, 62, 95, 126–128]. The degree of expression in amacrine cells should be considered when delivering regeneration-associated genes to RGCs, as these cells are known to influence the axon regeneration response of RGCs [120]. Our finding that the SYN promoter outperforms CMV in terms of transgene expression strength, and more targeted neuronal expression, is also consistent with studies comparing both promoters in adult rat retina [95] and in mouse and rat brain [45]. Expression by AAV2-SYN is however significantly different in the retina of macaque after intravitreal injection, as it failed to activate transgene expression in naive retina and expression was only seen after ganglion cell loss or vitreolysis [114]. This dramatically different expression pattern in non-rodents is therefore a crucial consideration for translation studies and warrants further investigation. Intravitreal injection of AAV2 containing SYN has however been used for electrophysiology and two-photon imaging of optic nerve axons in mice [127]. The AAV2 serotype and SYN promoter are an optimal combination for studying neural repair targets in rodents, and are an effective promoter choice to deliver *Cre* recombinase and regeneration-associated genes to RGCs when studying optic nerve regeneration [119, 129, 130].

### Cellular localisation of proteins following gene transfer

Consistent with a previous report [13], we show that intravitreal injection of AAV2 with the eGFP transgene results in the transduction of RGC and fluorescent fibres can be observed towards the LGN and the superior colliculus in the brain. eGFP is a soluble protein that homogenously fills the cytosol by diffusion [131, 132] revealing the full morphology of transduced cells. This axonal tracing could be valuable in studies examining axon regeneration after optic nerve injury where there is a need to visualise axon regeneration beyond the site of injury to successfully re-innervate their targets in the brain.

The spatial distribution of other proteins following gene transfer will depend on their biophysical properties. For instance, transcription factors will localise to the nucleus, endoplasmic reticulum-localised membrane proteins will be transported to this organelle, neurotrophic factors will be secreted extracellular, etc.

### Viral transduction in post-mortem human retina

The transduction in the post-mortem human retinal explants was low and predominantly favoured expression in retinal glia over neurons. This could be explained by the nature of the human tissue and the route of transduction. The rationale for performing the human retina explant experiment was to compare the efficacy of these vectors in a human system in the context of a whole system (i.e. not cultured human cells). In order to achieve this, ex vivo human tissue is required and unfortunately this comes with a number of caveats; i.e. impossible to perfectly age and genetically match tissue and there are many limitations when it comes to collection time post-death. As the eye is enucleated from the human donor this results in axotomy of all RGCs due to severing of the optic nerve in the enucleation process. As such, all RGCs will be going through neurodegenerative processes (i.e. Wallerian degeneration and active apoptosis). In addition to reduced RGC viability, the cellular uptake of viral vectors may be impaired in post-mortem tissue due to potential deficits in extracellular glycans and cell-surface receptors, endosomal trafficking and gene expression machinery. Therefore, all these constructs were tested in the context of a degenerating system and are not intended as a direct translational and therapeutic assessment. To test whether degenerating human retina contributes to the low viral transduction efficiency in the human tissue experiment, we performed the same experiments on mouse tissue where we can control the age, genetics, and time from death to culture. As expected, the mouse explant experiment closely mimics the human explant experiment unless performed on mice with delayed Wallerian degeneration (B6.BOla-*Wld*^*S*^ mice) which resulted in a higher RGC transduction efficiency despite axotomy.

Post-mortem human retina has previously been used to validate expression of viral constructs (AAV or lentivirus) including functional activity of neurons through the overexpression of channel rhodopsins [39, 128] or temperature-sensitive transient receptor potential channels [116]. It is worth noting that in these experiments RGC survival is not quantified (density appears comparable to the data presented here based on presented images of the GCL). It is likely that even in regions of high RGC density (such as the fovea), survival is limited, as noted by Sengupta et al. who recorded only six viable transduced cells from para-foveal retina (~1.6 × 1.6 mm area sampled). We specifically avoided the fovea in order to take multiple punches of retina where RGC density would be comparable between samples. As a result, RGC viability within this 7-day culture period is comparable between punches exposed to the different constructs suggesting that any potential differences in expression were due to the promotor sequence rather than internal variability in RGC survival and viability. It is unlikely that longer term culture conditions would increase AAV expression in RGCs, as RGCs are directly injured by axotomy from eye enucleation, and both rodent [133] and human [46] data supports an initial rapid loss of >50% of RGCs with only ~5% surviving long term (these are predominantly melanopsin expressing RGCs/ipRGCs [133]).

In addition, the inner limiting membrane also represents a substantial barrier to efficient transduction in the human [134, 135]. This is supported by the observation of high transduction at the retina punch border, where the retinal layers are exposed. In a clinical setting, an ILM peel is feasible to overcome this. Since the viral vectors were delivered to the surface of the explanted retina, expression of Müller glia end feet and astrocytes was predominant. Delivery in vivo with an ILM peel would likely yield more efficient transduction and expression, but long-term human efficacy and safety studies are required to address this.

## Conclusions

This study compares the transgene strength and cellular expression profile from five promoters in AAV2 4 weeks after intravitreal injection in mice. The conclusions of the study are summarised in Table 2. The CBA and CMV promoters may be good candidates for studies aiming to initiate moderate expression changes in the retina, while targeting a diverse range of cell types. The PGK promoter could be favourable when aiming to drive moderate expression in the neuronal cell population of the retina, including AII amacrine cells. The sCAG promoter has strong transgene expression and a diverse cellular tropism. This promoter would be suited to studies aiming to achieve optic nerve regeneration via secreted factors such as neurotrophins. The SYN promoter has strong transgene expression, and the high degree of RGC-specificity makes SYN a good promoter for direct gene transfer to RGCs. We’d therefore recommend selecting a SYN promoter to deliver regeneration-associated genes to RGCs to enhance their intrinsic axon regeneration capacity.

**Table 2:**
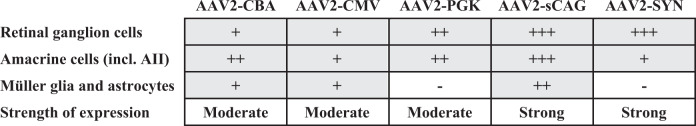
Cellular expression profile of AAV2 and five different promoters in mice after intravitreal injection.

Shaded squares indicate whether an AAV2 vector with the mentioned promoter could initiate transgene expression in the certain cell type in mice after intravitreal injection. The plus symbol (+) represents the level of transgene expression within the listed cell type. White squares with a minus symbol (−) represent no transgene expression was observed in the mentioned cell type. The transgene expression strength was classified as moderate or strong for each promoter. Rod bipolar- and horizontal cells are not shown in the table as they were poorly transduced when AAV vectors were delivered via intravitreal injection.

## Supplementary information

Supplementary table 1

Supplementary table 2

Supplementary figure legends

Supplementary figure 1

Supplementary figure 2

Supplementary figure 3

Supplementary figure 4

Supplementary figure 5

Supplementary figure 6

Supplementary figure 7

## Data Availability

The datasets generated and analysed during the current study are available from the corresponding authors on reasonable request.
