# Supplementary material for: Improving adeno-associated viral (AAV) vector-mediated transgene expression in retinal ganglion cells: comparison of five promoters
Source: Gene Ther. 2023 Jan 13;30(6):503–19. doi: 10.1038/s41434-022-00380-z (PMC10284706; doi:10.1038/s41434-022-00380-z)
Supplement: Supplementary file 2 — Supplementary table 2 [file 41434_2022_380_MOESM2_ESM.docx]

**Supplementary table 2.** Titre of adeno-associated viral vectors

| Adeno-associated viral vector | Production titre (qPCR) | Validation titre (QuickTiter kit) |
| --- | --- | --- |
| AAV2-CBA-eGFP (no Kozak) | 5.15 x 10^13^ | 4.03 x 10^12^ |
| AAV2-CBA-eGFP | 9.21 x 10^12^ | 3.77 x 10^12^ |
| AAV2-CMV-eGFP | 1.34 x 10^13^ | 3.75 x 10^12^ |
| AAV2-PGK-eGFP | 1.62 x 10^13^ | 4.35 x 10^12^ |
| AAV2-sCAG-eGFP | 2.27 x 10^13^ | 3.82 x 10^12^ |
| AAV2-SYN-eGFP | 2.72 x 10^12^ | 3.55 x 10^12^ |

The titre is shown as genome copies per millilitre (gc/mL). The production titre was determined by quantitative polymerase chain reaction and after the AAVs were matched to a titre of 2.5 x 10^12^ gc/mL an additional titre verification was done using a QuickTiter^TM^ AAV quantification kit. The latter confirms that the AAV titres are similar among the experimental groups in this study.
