# Supplementary material for: Improving adeno-associated viral (AAV) vector-mediated transgene expression in retinal ganglion cells: comparison of five promoters
Source: Gene Ther. 2023 Jan 13;30(6):503–19. doi: 10.1038/s41434-022-00380-z (PMC10284706; doi:10.1038/s41434-022-00380-z)
Supplement: Supplementary file 3 — Supplementary figure legends [file 41434_2022_380_MOESM3_ESM.docx]

**Supplementary figure 1** Validation that the AAV vector plasmids contain two inverted terminal repeat (ITR) sequences needed for efficient AAV packaging. Agarose gel loaded with AAV vector plasmids that underwent SmaI restriction enzyme digestion. The digestion resulted in three bands for each AAV vector plasmid, the upper band at the expected molecular weight of each linearized plasmid, and two lower bands as evidence of SmaI restriction enzyme digestion in both ITR sequences. The different agarose band sizes are due differences in the size of the full-length plasmids.

**Supplementary figure 2** AAV vector plasmids express eGFP in cultured HEK293T cells. Representative images are shown of cultured HEK293T cells two days post-transfection with AAV vector plasmids. eGFP was visible in all experimental groups, with the highest fluorescent intensity detected with the CBA, CMV, PGK, and sCAG promoters. SYN, being a neuronal-specific promoter, did not optimally express in HEK293T cells, but weak fluorescence could still be observed. eGFP signal is shown throughout without amplification using a GFP antibody.

**Supplementary figure 3** Intravitreal injection of AAV vectors for transgene delivery to the retina. 2μL of AAV vector was administered via Hamilton syringe connected to a 33-gauge needle at the 12 o’clock position. Care was taken to avoid penetration of the lens or damage to the vortex veins during the intravitreal injection. Injections were given slowly over 20 seconds to allow diffusion of the vector suspension. The eye appears slightly opaque during the injection, due to changes in vitreous volume, but colour rapidly returns prior to needle removal. Photographs taken via a handheld camera.

**Supplementary figure 4** Collection of neurological tissue for histology. Mouse eyes, brains and the optic chiasms were collected for further histological processing to visualise eGFP expression along the visual pathway. Photographs taken via a handheld camera.

**Supplementary figure 5** Inefficient transgene expression in Calbindin and PKC-α positive cells in the retina by five promoters when delivered via AAV2 and intravitreal injection. eGFP expression (green) and DAPI staining (blue) together with Calbindin (A, purple), PKC-α (B, cyan) staining in retinal cross-sections of mice injected with AAV2-CBA-eGFP, AAV2-CMV-eGFP, AAV2-PGK-eGFP, AAV2-sCAG-eGFP, and AAV2-SYN-eGFP. Images were taken using an epifluorescent microscope with identical microscope settings between the experimental groups per investigated cell-type. eGFP is shown throughout without amplification using a GFP antibody. Abbreviations: GCL, ganglion cell layer; INL, inner nuclear layer; ONL, outer nuclear layer.

**Supplementary figure 6** RGC viability in post-mortem human retinal explants. (A) Quantification of the number of RGCs in non-transduced post-mortem human punches at 0- and 7-DEV. (B) Quantification of the number of RGCs in 7 DEV post-mortem human punches that were transduced with AAV2-CBA-eGFP, AAV2-CMV-eGFP, AAV2-PGK-eGFP, AAV2-sCAG-eGFP, and AAV2-SYN-eGFP. This figure illustrates that the biggest source of variance in RGC viability is between post-mortem human retinas, not between AAV vectors. Abbreviation: DEV, days *ex vivo*.

**Supplementary figure 7** Expression overview in a post-mortem human retinal punch that received a droplet of AAV2-CMV-eGFP *ex vivo*. Staining for the transgene eGFP (green) is shown and transduction was predominantly at the borders of the retinal punch. Images was taken using a confocal microscope.
