# Supplementary material for: Improving adeno-associated viral (AAV) vector-mediated transgene expression in retinal ganglion cells: comparison of five promoters
Source: Gene Ther. 2023 Jan 13;30(6):503–19. doi: 10.1038/s41434-022-00380-z (PMC10284706; doi:10.1038/s41434-022-00380-z)

Validation of ITR sites in plasmids

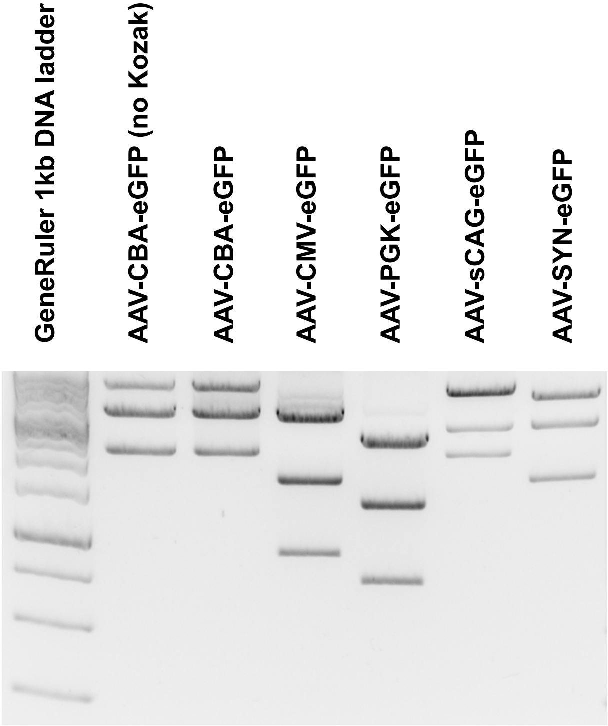

Supplement: Supplementary file 4 — Supplementary figure 1 [file 41434_2022_380_MOESM4_ESM.pdf]
