# Supplementary material for: Improving adeno-associated viral (AAV) vector-mediated transgene expression in retinal ganglion cells: comparison of five promoters
Source: Gene Ther. 2023 Jan 13;30(6):503–19. doi: 10.1038/s41434-022-00380-z (PMC10284706; doi:10.1038/s41434-022-00380-z)

Intravitreal injection of AAV vectors for transgene delivery to the retina

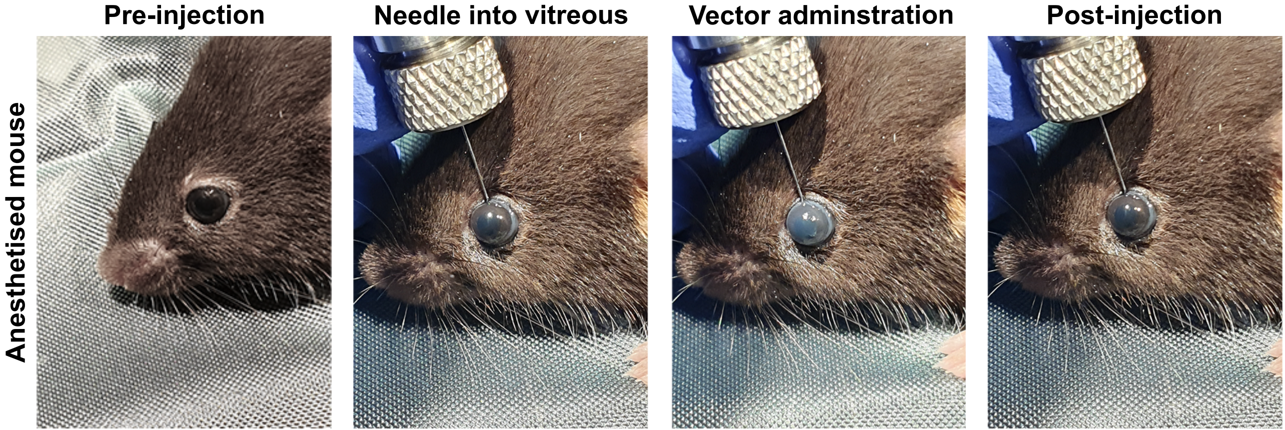

Supplement: Supplementary file 6 — Supplementary figure 3 [file 41434_2022_380_MOESM6_ESM.pdf]
