# Supplementary figures and images for: Improving adeno-associated viral (AAV) vector-mediated transgene expression in retinal ganglion cells: comparison of five promoters
Source: Gene Ther. 2023 Jan 13;30(6):503–19. doi: 10.1038/s41434-022-00380-z (PMC10284706; doi:10.1038/s41434-022-00380-z)

# Inefficient transgene expression in Calbindin- and PKC- $\alpha$ positive cells

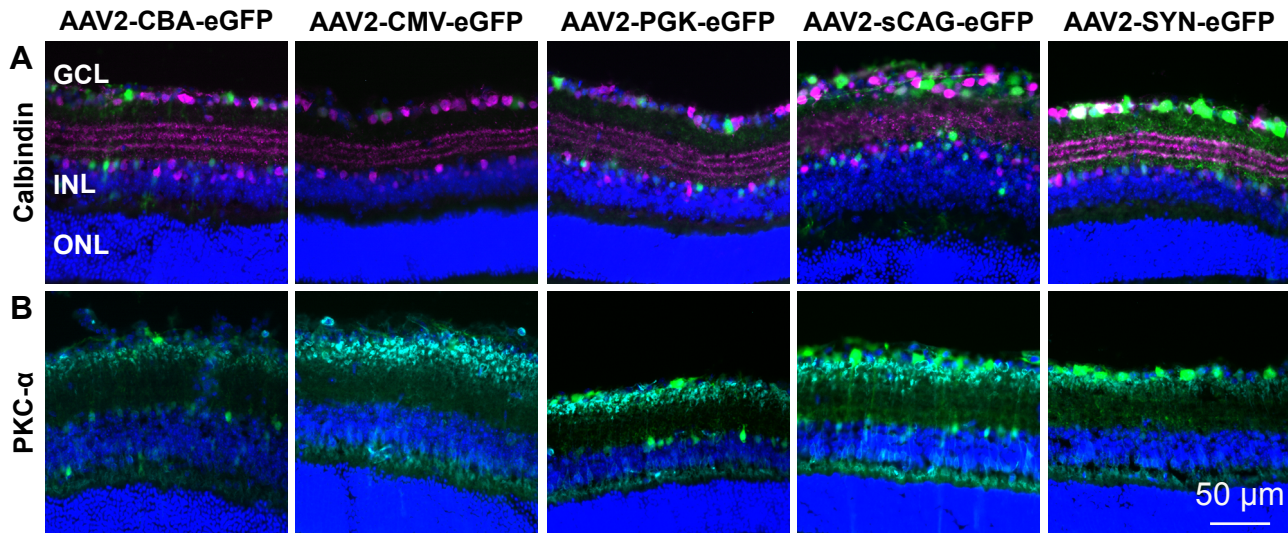

Supplement: Supplementary file 8 — Supplementary figure 5 [file 41434_2022_380_MOESM8_ESM.pdf]

# RGC viability in post-mortem human retinal explants

## i. overtime

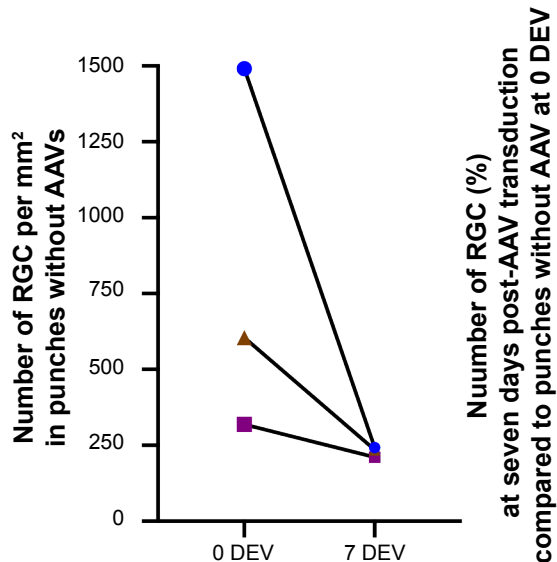

## ii. per viral vector per donor

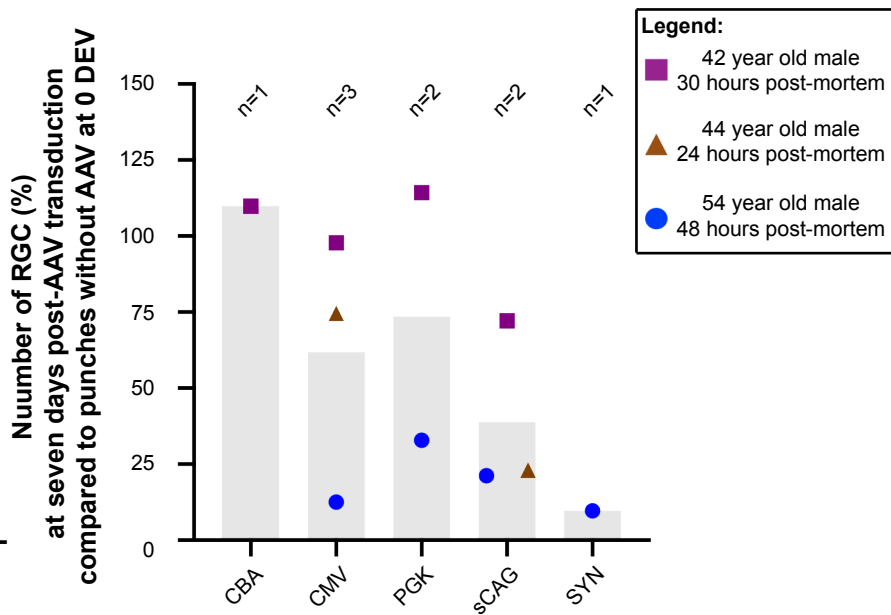

Supplement: Supplementary file 9 — Supplementary figure 6 [file 41434_2022_380_MOESM9_ESM.pdf]

# AAV expression overview

Post-mortem human retinal punch

AAV2-CMV-eGFP

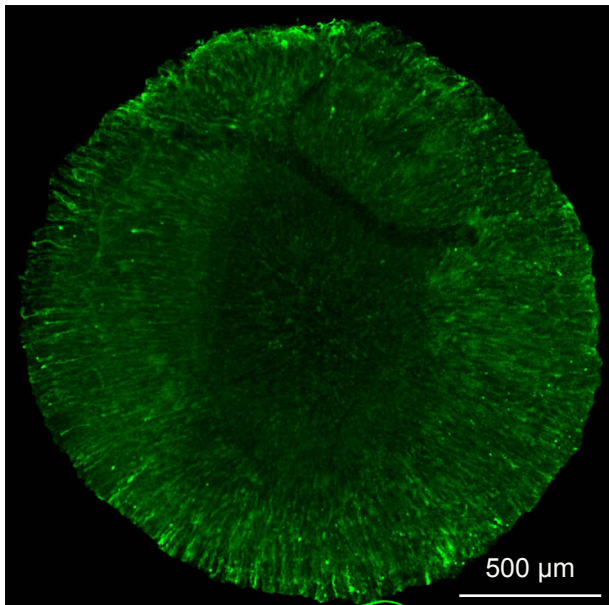

Supplement: Supplementary file 10 — Supplementary figure 7 [file 41434_2022_380_MOESM10_ESM.pdf]
